# Gut microbiota: a novel strategy affecting atherosclerosis

**DOI:** 10.1128/spectrum.00482-24

**Published:** 2025-07-10

**Authors:** Lingshuang Yang, Juan Yang, Tiantian Zhang, Xinqiang Xie, Qingping Wu

**Affiliations:** 1 Modern Industrial College of Traditional Chinese Medicine and Health, Zhejiang Lishui Service Platform for Technological Innovations in Traditional Chinese Medicine Industry, Lishui Institute of Traditional Chinese Medicine, Lishui University 117807, Lishui, Zhejiang, China; 2 College of Food Science, South China Agricultural University 12526, Guangzhou, Guangdong, China; 3 National Health Commission Science and Technology Innovation Platform for Nutrition and Safety of Microbial Food, Guangdong Provincial Key Laboratory of Microbial Safety and Health, State Key Laboratory of Applied Microbiology Southern China, Institute of Microbiology, Guangdong Academy of Sciences 514144, Guangzhou, Guangdong, China; National Institutes of Health, Bethesda, Maryland, USA

**Keywords:** gut microbiota, probiotics, atherosclerosis, prebiotics, metabolites

## Abstract

Atherosclerosis is a common chronic inflammatory cardiovascular disease affecting both coronary and peripheral arteries, which is influenced by multiple factors. It is increasingly evident that gut microbes and their byproducts play a crucial role in the development of atherosclerosis. The most representative feature of microbial dysbiosis in coronary artery disease patients is the reduction of the abundance of the phylum Bacteroidetes and the increase of the abundance of the phylum Firmicutes, which may cause changes in functional genes and metabolites. The gut microbiota and its metabolites influence the early, intermediate, and late stages of atherosclerosis mainly by inhibiting or promoting inflammatory responses. In addition, the reshaping of gut microbiota through probiotics, prebiotics, and fecal microbiota transplantation (FMT) is discussed as an alternative to traditional therapeutic methods. By summarizing how gut microbiota and their metabolites affect the process of atherosclerosis, we can better understand the complex relationship between gut microbiota and atherosclerosis.

**IMPORTANCE:**

Atherosclerosis is an inflammatory cardiovascular disease, making it crucial to understand its pathogenesis and develop effective treatments. This review thoroughly examines the literature, emphasizing the gut microbiome as a key factor influencing atherosclerosis. It also explores how the gut microbiota and its metabolites impact the primary, intermediate, and advanced stages of atherosclerosis and proposes that remodeling the gut microbiota is a promising strategy for improving atherosclerosis.

## INTRODUCTION

Atherosclerosis is the process of plaque accumulation in the arteries, which results in the constriction of arteries and a heightened susceptibility to heart attack and stroke. It represents a significant contributor to cardiovascular diseases in Western nations and stands as the primary cause of mortality in the United States (1). It is well known that diseases such as hyperlipidemia, hypertension, obesity, metabolic syndrome, and diabetes are considered to be associated with the development of atherosclerosis. Other factors are also crucial in the progression of atherosclerosis, such as age, diet, psychology, and gut microbiota. The ratio of bacteria to human cells in the gut microbiota is approximately 1.3:1, with a diverse range of bacterial species ranging from 500 to 1,000. The most abundant phyla in the gut microbiota are Bacteroidetes and Firmicutes, which play important roles in regulating metabolic functions and maintaining immune homeostasis (2). Numerous studies have reported alterations in the gut microbiota caused by inflammatory bowel disease, asthma, obesity, metabolic disorders, and cardiovascular diseases (CVD) (3–5). There are often differences in the gut microbiota between patients with atherosclerosis and healthy individuals. The abundance of Enterobacteriaceae, oral bacteria, and *Clostridia* is significantly increased in the gut of patients with coronary atherosclerosis. Moreover, there is a significant positive correlation between changes in *Streptococcus* abundance and blood pressure, as well as a positive correlation between changes in Enterobacteriaceae abundance and cardiac markers (6). The gut microbiota can affect atherosclerosis by influencing the production of proinflammatory cytokines and metabolites. Classical proinflammatory signaling pathways, such as the TLR2/NF-κB and TLR4/NF-κB pathways, are involved in the inflammatory process caused by gut microbiota infections. Therefore, understanding the role of gut microbiota in atherosclerosis development is crucial for developing effective treatments (7). The aim of this review is to evaluate the feasibility of gut microbiota intervention as a potential way to improve atherosclerosis. First, this review provides a detailed analysis of the potential role of gut microbiota in the development of atherosclerosis. Furthermore, it discusses the molecular pathways and mechanisms through which gut microbiota and their metabolites may impact atherosclerosis. Finally, the review concludes by discussing the use of probiotics, prebiotics, engineered bacteria, and fecal microbiota transplantation (FMT) as methods to reshape the gut microbiota to alleviate the progression of atherosclerosis.

## RESULTS AND DISCUSSION

### Atherosclerosis formation

In the early stages of atherosclerosis, activated endothelial cells (ECs) release cytokines and chemokines, which promote the recruitment and aggregation of monocytes and macrophages. These macrophages take up modified LDL cholesterol, known as “bad” cholesterol, and break it down for removal from the blood (8, 9). The binding of circulating LDL surface apolipoprotein B (ApoB) to LDL receptors on ECs triggers endocytosis of LDL into the inner membrane, resulting in the formation of foam cells (8, 10). However, LDL can be oxidized to form oxidized LDL (oxLDL) through enzymatic attack or reaction with reactive oxygen species (ROS) (11). The accumulation of oxLDL in the arterial wall triggers the expression of VCAM-1, a cell adhesion molecule, and the production of M-CSF, which promotes the differentiation of monocytes into macrophages (11). The foam cells produced by the accumulation of oxLDLs in the arterial wall also exhibit the expression of Toll-like receptor (TLR), pattern recognition receptor (PRR), and scavenger receptor. These receptors are important for the recognition of pathogens and other foreign substances and help initiate an immune response to protect the body from further damage (11). In addition, vascular smooth muscle cells (VSMCs) undergo proliferation and migration toward neighboring areas, transitioning into various cell phenotypes: macrophage-like, foam cell, mesenchymal stem-like, myofibroblast-like, and osteochondral-like. The differentiation of VSMCs into “inflammatory” macrophage-like cells may contribute to the instability of atherosclerotic lesions, whereas their transition into “synthetic” fibrotic VSMCs could stabilize lesions by increasing the thickness of protective fibrous caps. Whether these phenotypic changes of VSMCs predominantly exert protective or detrimental effects on atherosclerosis remains uncertain and may depend on the microenvironment. Thus, cells originating from VSMCs may play a dual role, enhancing both plaque stability and susceptibility to rupture (12, 13).

**Fig 1:**
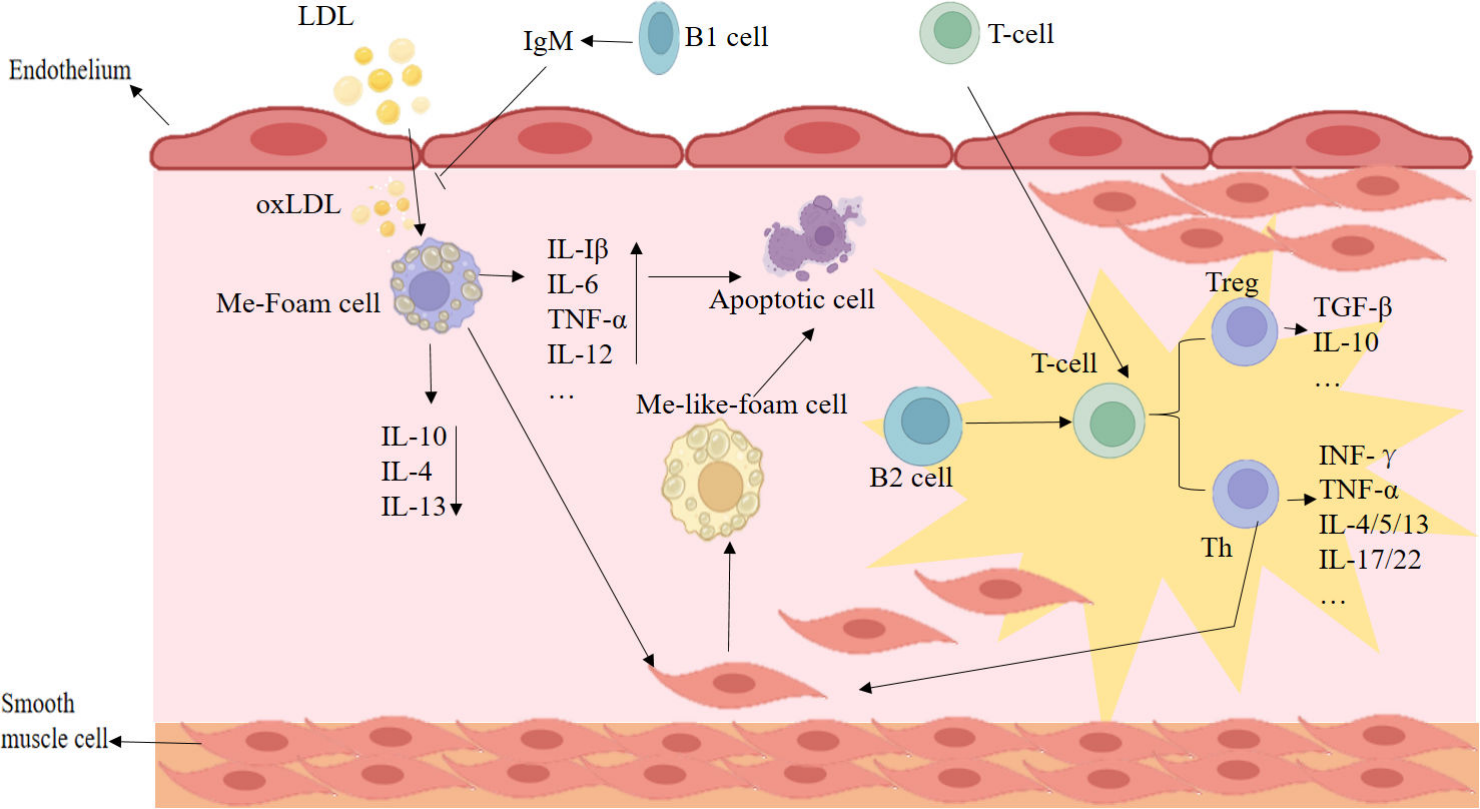
Main formation mechanism of atherosclerosis. IL-1β, interleukin-1β; IL-6, interleukin-6; IL-2, interleukin-2; IL-4, interleukin-4; IL-5, interleukin-5; IL-12, interleukin-12; IL-13, interleukin-13; IL-10, interleukin-10; IL-22, interleukin-22; IL-17, interleukin-17; TNF-α, tumor necrosis factor-α; TGF-β, transforming growth factor-β; IFN-γ, interferon gamma; oxLDL-C, oxidized low-density lipoprotein cholesterol; VSMCs, vascular smooth muscle cells.

Finally, T cells are activated by interacting with antigen-presenting cells (APCs) such as dendritic cells (DCs) and macrophages, resulting in the production of cytokines like INF-γ, which further contributes to unstable plaque development (14) (Fig. 1). Unstable plaques can rupture and trigger thrombus formation, adding further disadvantages. To maintain cholesterol balance, the body employs internal balancing mechanisms such as reverse cholesterol transport (RCT). RCT is a mechanism that aids in the elimination of surplus cholesterol from peripheral tissues, reducing the risk of unstable plaque and thrombus formation. Liver-produced bile acids (BAs) assist in excreting cholesterol from the body, preventing its accumulation (15, 16). High-density lipoprotein (HDL), primarily carrying cholesterol to the liver, relies on apolipoprotein A1 (ApoA1) and regulators like liver X receptors (LXRs) α and β, ATP-binding cassette transporter A1 (ABCA1), and ATP-binding cassette transporter G1 (ABCG1). They ensure efficient cholesterol transportation to the liver for metabolism (16). By upregulating the expression of cholesterol transporters like ABCA1 and ABCG1, macrophages effectively eliminate oxLDL from cells and facilitate its transportation to the liver for excretion via bile acids (16). This outflow of cholesterol from foam cells is vital for the body’s defense against atherosclerosis as it reduces the buildup of fatty deposits in the arteries and reduces the likelihood of experiencing a heart attack and stroke.

### Primary factors influencing the formation of atherosclerosis

**Fig 2:**
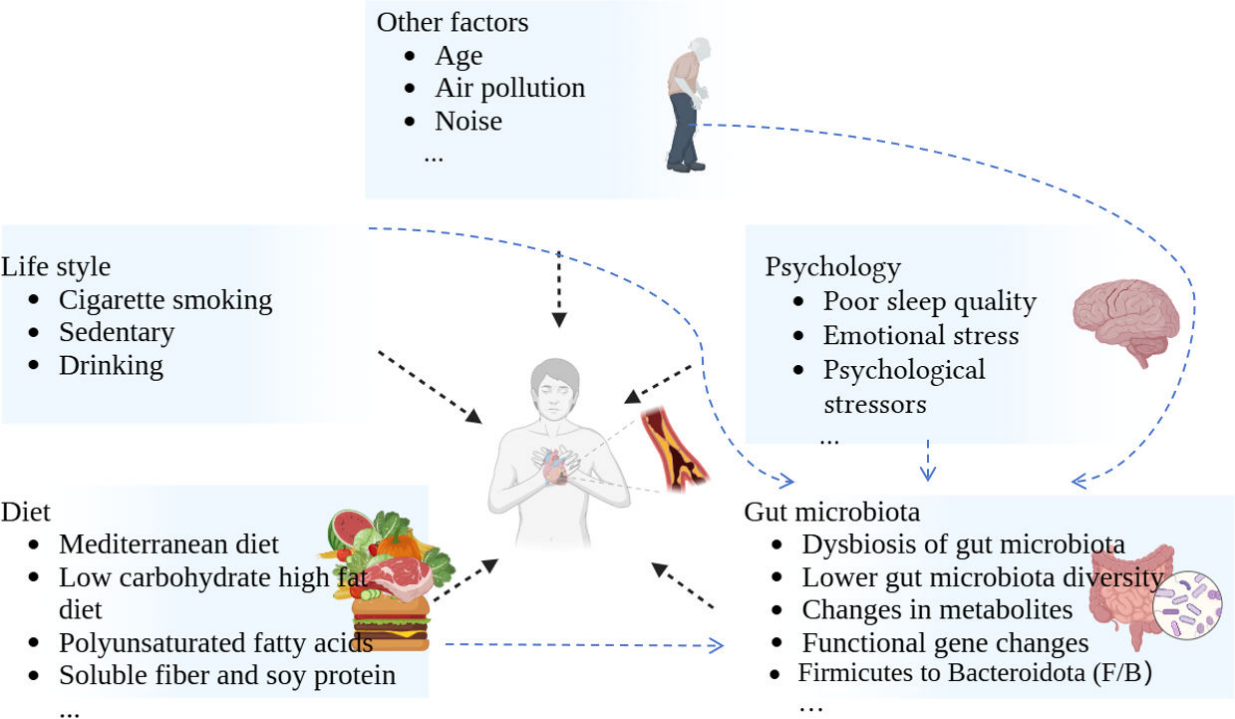
Key factors affecting the formation of atherosclerosis.

The development of atherosclerosis is highly complex and can be broadly categorized into early, intermediate, and advanced stages. It is well known that conditions such as hyperlipidemia, hypertension, obesity, metabolic syndrome, and diabetes are associated with the progression of atherosclerosis (17). Research evidence suggests that visceral and ectopic fat may contribute to the development of atherosclerosis and increase the risk of cardiometabolic diseases (18, 19). Regardless of hyperlipidemia, diabetes, and accompanying changes in blood glucose levels, abnormal lipid profiles, and other metabolic alterations, these conditions are closely associated with the pathogenesis of atherosclerosis at almost every step of its progression (17). The exact causes of atherosclerosis are influenced by multiple factors (20). This review primarily focuses on the following: diet, psychological, lifestyle, gut microbiota, and other factors and discusses their impact on the development of atherosclerosis (Fig. 2).

#### Diet

Diet has a significant impact on the development of atherosclerosis. First, a clinical cohort study showed that long-term consumption of a Mediterranean diet rich in extra-virgin olive oil can reduce the intima-media thickness of both common carotid arteries (IMT-CC) and the height of carotid artery plaques compared to a low-fat diet, thus reducing the severity of atherosclerosis (21). Research suggests that compared to the control group, consuming less than 20 g of carbohydrates per day leads to a 44% increase in LDL-C levels. However, there is significant variation in individual responses to a low-saturated fat diet (22). Research has indicated a positive correlation between polyunsaturated fatty acids (PUFAs) and the progression of atherosclerosis. For every 5% increase in n-6 PUFA energy intake, there is an increase of one copy of the SREBP1 genotype, resulting in a 0.21 mm increment in the mean minimum coronary artery diameter in females (23). With omega-3 PUFA supplementation, a reduction in the progression of carotid intima-media thickness was observed (24). Second, the combination of soluble fiber and soy protein has been shown to significantly reduce LDL-C by 20% after 2 months (25). Soluble fiber is believed to bind with bile acids in the intestinal lumen, forming micelles. Through physical entrapment, this process leads to an increased synthesis of bile acids, reduced hepatic cholesterol content, upregulation of LDL receptors (LDLr), and an increased rate of LDL clearance (26, 27). Third, plant sterols are derived from vegetable oils, and clinical trial evidence shows that plant sterols/sterols can reduce LDL-C by 6%–15% at a dose of 2–3 g/ day。The mechanism is that plant sterols compete with cholesterol for space within the intestinal bile salt micelles, thereby reducing the absorption of cholesterol (28). An increase in the amount of plant sterols in the gut reduces the micellar solubility of cholesterol and decreases the amount of cholesterol that can be absorbed (29). Another mechanism involves their interaction with ATP-binding cassette transporters in intestinal cells, which guides the cholesterol back to the intestines. Lastly, there are minerals and antioxidants present in certain fruits that also influence the development of atherosclerosis. Vitamin C, for example, protects the endothelium by enhancing eNOS (endothelial nitric oxide synthase) function (30). Resveratrol is a polyphenol found in the skin of grapes, berries, and peanuts. It possesses antioxidant properties (resveratrol: French paradox revisited), inhibits platelet aggregation and lowers LDL-C levels (31)，and reduces atherosclerosis. The mechanism is to increase the production of nitric oxide synthase (32) and improve mitochondrial function by activating AMP-activated protein kinases (AMPK) and sirtuins (33).

#### Lifestyle

Cigarette smoking (CS) increases the incidence of myocardial infarction (MI) and fatal coronary artery disease (CAD) in both men and women. Even low-tar cigarettes and smokeless tobacco are increasing the risk of cardiovascular events compared to non-smokers, and some recent experimental clinical studies have shown a nonlinear relationship between cigarette smoke exposure and risk (34). The exact toxic components of cigarette smoke and the mechanisms underlying the association between cigarette smoke and cardiovascular dysfunction are still largely unclear. However, cigarette smoke exposure increases inflammation, thrombus formation, and oxidation of low-density lipoprotein cholesterol (LDL-C) (34). Furthermore, sedentary behavior and alcohol consumption are also detrimental lifestyle factors that can affect the progression of atherosclerosis (35). In conclusion, lifestyle changes may be able to reduce the overall cardiovascular disease risk.

#### Psychology

It is well known that psychological stress is associated with increased atherosclerosis (36). In a diverse sample of over 1,600 individuals, poor sleep quality was found to increase the severity of atherosclerosis by elevating inflammation-related white blood cell counts (neutrophils and monocytes), even after accounting for other common risk factors. Therefore, improving sleep quality may serve as a preventive strategy for reducing inflammation and the risk of atherosclerosis (37). Emotional stress is associated with an increased risk of cardiovascular disease. The mechanisms underlying this are partly mediated through increased amygdalar activity in the brain, which leads to enhanced bone marrow activity and arterial inflammation (38). Studies have shown a correlation between the severity score of metabolic syndrome (MetS) and the risk of depression (39). Psychological stressors, such as public speaking and mental arithmetic tasks, can significantly induce increased levels of systemic interleukin-6 and C-reactive protein (CRP) (40). To some extent, inflammation is considered a component of the physiological stress response. Clinical research suggests that the biological pathways may be pathological, with stress activating the sympathetic nervous system (SNS), hypothalamic-pituitary-adrenal (HPA) axis, and renal renin-angiotensin system, leading to the release of various stress hormones such as catecholamines, corticosteroids, glucagon, and growth hormone. The SNS, HPA axis, and renin-angiotensin system can also cause an increase in homocysteine levels, which subsequently leads to heightened cardiovascular activity, endothelial dysfunction, and induction of endothelial cell adhesion molecules (41). Adhesion molecules can recruit immune/inflammatory cells and facilitate their adhesion to the arterial wall. Psychological stress and acute-phase reactants promote inflammatory responses, including macrophage activation, formation of free radicals, lipid modification, foam cell formation, and the occurrence of thrombotic events that can lead to stable or unstable plaques.

#### Gut microbiota

The gut microbiota plays a crucial role in the initiation and progression of atherosclerosis. In recent years, numerous studies have indicated that patients with atherosclerosis exhibit alterations in gut microbiota. Researchers have started investigating the relationship between atherosclerosis and gut microbiota, as shown in Table 1. The composition of bacterial abundance groups (CAGs), specifically *Roseburia*, *Klebsiella*, *Clostridium IV*, and *Ruminococcaceae*, undergoes characteristic changes at different stages of coronary artery disease (CAD) (42). In their metagenomic study, Liu et al. found decreased abundance of *Bacteroides xylanisolvens*, *Odoribacter splanchnicus*, *Eubacterium eligens*, *Roseburia inulinivorans*, and *Roseburia intestinalis* in patients with atherosclerosis. These patients were also found to have deficiencies in starch metabolism (glycogenolysis) pathway V, sugar fermentation pathway III, CDP-diacylglycerol biosynthesis, and folate conversion pathways compared to healthy individuals (43). Another study revealed a significant increase in the abundance of *Prevotella* and a significant decrease in the abundance of *Clostridium* and *Faecalibacterium* in Polish male patients with atherosclerosis. On the other hand, Chinese males exhibited a significant increase in the abundance of *Streptococcus* and *Escherichia*, along with a significant decrease in the abundance of *Bacteroides* and *Prevotella*(44). Among the numerous pieces of evidence presented in Table 1, it is consistently demonstrated that the Firmicutes-to-Bacteroidota (F/B) ratio is elevated in the gut microbiota of patients. This ratio serves as a potential indicator. The aforementioned evidence suggests that atherosclerosis leads to dysbiosis in the gut microbiota, with possible variations among countries and ethnicities. Furthermore, atherosclerotic plaques themselves constitute a microbial environment containing bacteria such as *Streptococcus, Pseudomonas*, *Klebsiella*, *Fusobacterium*, and *Chlamydia pneumoniae (*45, 46). Studies have indicated that pathogenic bacteria from the oral or gut microbiome can directly infect the vascular wall or induce autoimmune inflammatory responses through molecular mimicry, leading to increased susceptibility to plaque formation in the blood vessels (47). Furthermore, during the development of atherosclerosis, there is increased diversity observed in the phage community. In the future, greater attention will be given to studying the abundance fluctuations of phages.

**TABLE 1:** Evidence that gut microbes influence atherosclerosis^*a*^

	Population	Country	Age (years)	Sample size	Disease	Sequencing method	Higher abundance in CAD	Lower abundance in CAD	Alpha diversity in CAD	Ref.
Karlsson et al., 2017	Human	Sweden	55–72	*n* = 12 CAD, *n* = 13 controls	Atherosclerosis	Metagenome	*Collinsella* genus, Enterobacteriaceae, Streptococcaceae, and *Klebsiella* spp.	*Eubacterium*, *Roseburia*, and Ruminococcaceae spp.	/	(42)
Liu et al., 2020	Human	Sweden and China	/	/	Atherosclerosis	Metagenome	/	*Bacteroides xylanisolvens*, *Odoribacter* *splanchnicus*, *Eubacterium eligens*, *Roseburia inulinivorans*, and *Roseburia intestinalis*	/	(43)
Cui et al., 2017	Human	China	68.3 ± 9.5	*n* = 29 CAD, *n* = 35 controls	CAD verified by coronary angiography	16S rRNA	Firmicutes phylum	Bacteroidetes	Higher	(48)
Jie et al., 2017	Human	China	40–80	*n* = 218 CAD, *n* = 187 controls	Atherosclerosis cardiovascular disease (ACVD)	Metagenomics	*Streptococcus* and *Escherichia*; *Lactobacillus* *salivarius*, *Solobacterium moorei*, and *Atopobium parvulum*, *Ruminococcus gnavus*, and *Eggerthella lenta*	*Bacteroides* and *Prevotella*, *Roseburia intestinalis* and *Faecalibacterium prausnitzii*, *Bacteroides* spp., *Prevotella copri,* and *Alistipes shahii*	/	(6)
Zhu et al., 2018	Human	China	63.5	*n* = 70 CAD, *n* = 98 controls	CAD verified by coronary angiography	16S rRNA	*Escherichia-Shigella* and *Enterococcus*	*Faecalibacterium*, *Subdoligranulum, Roseburia,* and *Eubacterium rectale*	Lower	(49)
Feng et al., 2016	Human	China	/	*n* = 59 CHD, *n* = 43 controls	Coronary heart disease (CHD)	Metagenome	*Clostridium* sp. *HGF2, Streptococcus* sp. *M334,* and *Streptococcus* sp. *M143*	/	/	(50)
Yoshida et al., 2018	Human	Japan	/	*n* = 30 CHD, *n* = 30 controls	Coronary artery disease (CAD)	16S rRNA	*Faecalibacterium prausnitzii* and *Prevotella copri*	*Bacteroides vulgatus* and *Bacteroides dorei*	No	(51)
Liu et al., 2019	Human	China	49–72	*n* = 161 CHD, *n* = 40 controls	CAD	16S rRNA	/	*Bacteroides xylanisolvens, Odoribacter splanchnicus, Eubacterium eligens, Roseburia inulinivorans,* and *Roseburia intestinalis*	No	(52)
Panyod et al., 2023	ApoE−/− mice			*n* = 8	Atherosclerosis	16S rRNA	*Enterorhabdus, Romboutsia, Proteus, Eubacterium nodatum, Escherichia-Shigella, Eubacterium coprostanoligenes, Parasutterella, Muribaculum,* and *Enterococcus*	*Bifidobacterium* and *Alistipes*	Lower	(53)
Liu et al., 2021	ApoE−/− mice			*n* = 6	Atherosclerosis	16S rRNA	*Proteobacteria* and *Firmicutes; Romboutsia* and *Lactococcus*	*Bacteroidetes* and *Actinobacteria;* *Faecalibaculum, Bifidobacterium, Bacteroides, Parabacteroides, Alloprevotella, Alistipes, Odoribacter,* and *Allobaculum*	Lower	(54)
Xie et al., 2022	ApoE−/− mice			*n* = 6	Atherosclerosis	16S rRNA	Firmicutes; *Faecalibaculum*, *Oscillibacter*, *Eubacterium_coprostanoligenes* _group, and *Blautia*	*Muribaculaceae*, *Lactobacillus*, *Ileibacterium*, and *Bifidobacterium*	Lower	(55)
Yang et al., 2022	ApoE−/− mice			*n* = 5	Atherosclerosis	16S rRNA	Firmicutes to Bacteroidota (F/B); Lactobacillaceae; *Lactobacillus* and *Helicobacter*	Lachnospiraceae; *Roseburia*	Lower	(56)
Liu et al., 2019	ApoE−/− mice			*n* = 10	Atherosclerosis	16S rRNA	Firmicutes to Bacteroidota (F/B); Verrucomicrobia; Ruminococcaceae and Bacteroidaceae; *Bacteroides* and *Akkermansia*	Rikenellaceae	/	(57)
Zhu et al., 2020	ApoE−/− mice		8 ± 1 weeks old	*n* = 6	Atherosclerosis	16S rRNA	Firmicutes; *Lactobacillus*	Bacteroidetes; *Bifidobacterium*	Lower	(58)

^*a*^ / indicates that no relevant information was described in the included literature.

Current research suggests a contradictory role of the Lactobacillaceae family in atherosclerosis. Lactobacillaceae is generally regarded as a probiotic that can prevent various diseases. However, it has been reported that its abundance is decreased in patients with acute coronary syndrome (59). Huang et al. (60) reported that a Lactobacillaceae member, *Lactobacillus acidophilus* ATCC 4356, prevents the progression of atherosclerosis in ApoE–/– mice by inhibiting intestinal cholesterol absorption. However, Chen et al. (61) reported that *L. casei* accelerated atherosclerosis in mouse models of Kawasaki disease. In the Tampere sudden death study, *Lactobacillaceae* was positively correlated with the area of coronary atherosclerotic plaque in patients, and *Lactobacillaceae* DNA was amplified in coronary plaque, suggesting intestinal bacterial translocation. Due to strain specificity, there may be differences in gut microbes in different populations, so more studies are needed to prove the specific functional characteristics of these bacteria in the future. Some of these bacteria have the ability to regulate the metabolic pathways of the host, impacting atherosclerosis through processes like short-chain fatty acid (SCFA), TMAO, LPS, taurine, sphingolipid, ceramide, and benzene metabolism. Researchers have developed a disease classifier based on the varying levels of microorganisms and metabolites, enabling the differentiation of CAD cases from controls. Moreover, this classifier has the capability to accurately discriminate between stable coronary syndromes and acute coronary syndromes (52). Gut microbiota has the ability to stimulate the immune system, serving as a defense mechanism against pathogens. Additionally, it can modulate the expression of vascular microRNA-204 (Mir-204), which is controlled by the microbiome from a distance. This regulation can impact endothelial function by targeting Sirtuin 1 lysine deacetylase (Sirt1). These findings suggest that the gut microbiota can influence the development and occurrence of atherosclerosis through both metabolically independent and metabolically dependent pathways (62). In conclusion, the microbiota plays a crucial role in regulating systemic immunity and metabolism, primarily through the production of microbial metabolites.

#### Other factors

Age plays a critical role in the deterioration of cardiovascular function, leading to an increased risk of CVD in older individuals, particularly atherosclerotic cardiovascular diseases (63). As age increases, arterial stiffness tends to increase, resulting in reduced arterial compliance and higher pulse wave velocity. The increased shear stress associated with these changes exacerbates the formation of atherosclerotic plaques. Atherosclerotic peripheral arterial disease (PAD) affects at least 4.5% of adults aged 40 and above, and as many as 8%–12% of individuals aged 65 and above in the United States (64). Age ≥ 45 for men and ≥55 for women is considered a major risk factor for developing CHD, and in the United States, over 80% of CHD-related mortality occurs in patients older than 65 years of age (65). Increased conduit artery wall thickness is an independent predictor of cardiovascular diseases (66, 67). Advanced age is associated with conduit artery wall thickening, which is a manifestation of atherosclerosis (68, 69). There is a growing realization that the altered assembly, structure, and dynamics of the gut microbiota actively participate in the aging process (70). This further suggests that gut microbes affect the process of atherosclerosis. Furthermore, environmental factors such as air pollution, noise, and UV radiation also influence the development of atherosclerosis (71).

### Gut microbes influence the pathway of atherosclerosis formation

The gut microbiota and its metabolites influence various stages of atherosclerosis, including the initial, intermediate, and advanced phases. In the initial phase, it involves ECs and macrophages (M). In the intermediate phase, it involves vascular smooth muscle cells (VSMCs). In the advanced phase, it involves T cells, B cells, and DCs. The pathways through which the gut microbiota and its metabolites impact each stage of atherosclerosis exhibit variations and require further discussion.

#### Effects of gut microbiota in the early stages of atherosclerosis

**Fig 3:**
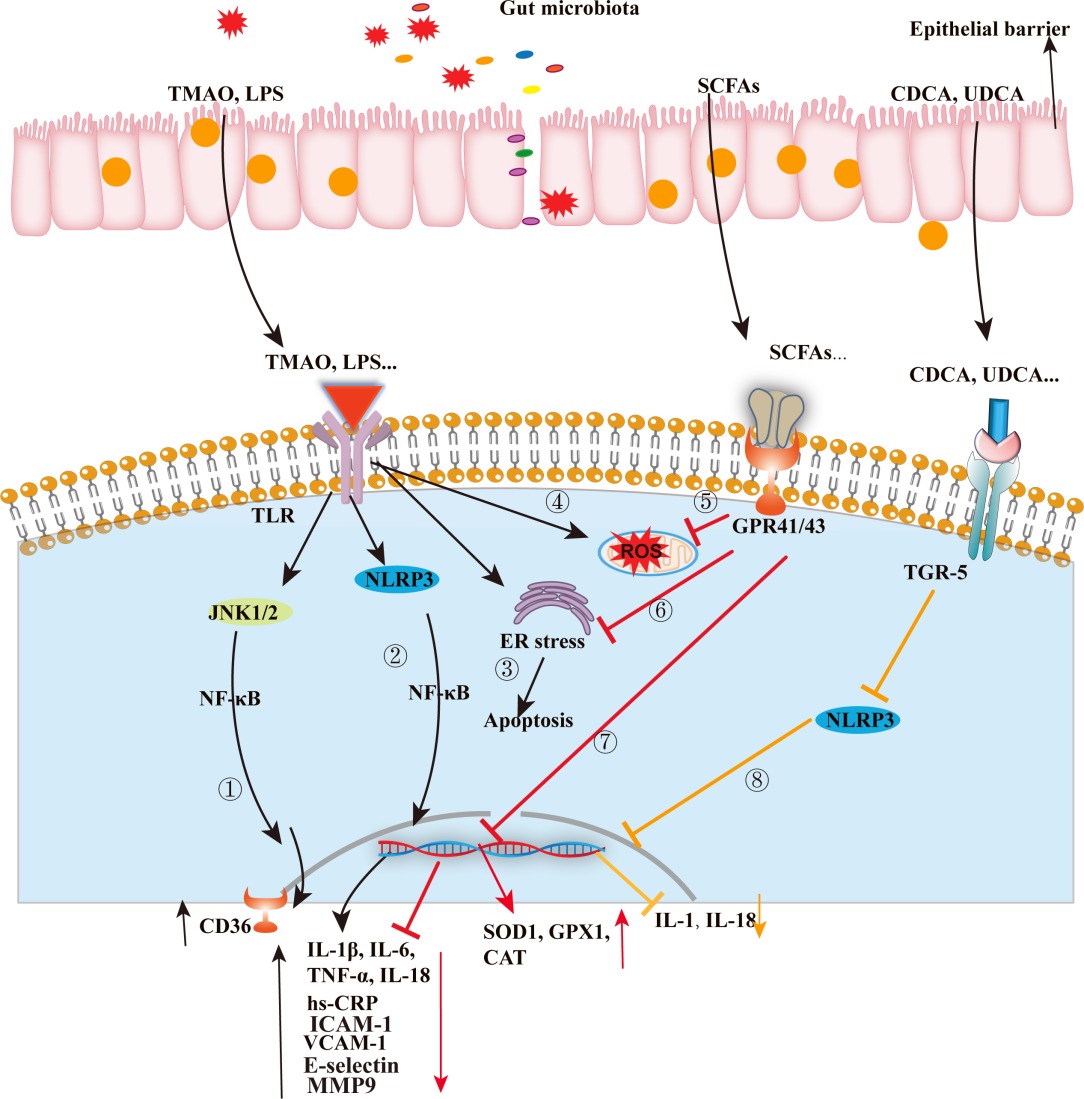
The potential pathway by which gut microbes and metabolites influence atherosclerosis. ①②③④TMAO, LPS, and other metabolites primarily regulate the signaling pathways including JNK, NLRP3, ER-stress, and mitochondrial oxidation, resulting in the production of inflammatory factors and adhesion molecules that exacerbate disease progression. On the contrary, metabolites such as short-chain fatty acids (SCFAs) yield opposite effects⑤⑥⑦. Additionally, bile acids like CDCA and UDCA regulate the TGR5 signaling pathway to suppress inflammatory responses⑧. Notes: SCFAs, short chain fatty acids; TMAO, trimethylamino oxide; LPS, lipopolysaccharide; CDCA, chenodeoxycholic acid; UDCA, ursodesoxycholic acid; IL-1β, interleukin-1β; IL-6, interleukin-6; IL-18, interleukin-18; IL-1, interleukin-1; TNF-α, tumor necrosis factor-α; hs-CRP, high-sensitivity C-reactive protein; VCAM-1, vascular cell adhesion molecule-1; MMP9, matrix metalloprotein9; SOD1, superoxide dismutase1; GPX1, glutathione peroxidase 1; CAT, catalase; CD36, cluster of differentiation 36; NF-κB, nuclear factor-k-gene binding; ER stress, endoplasmic reticulum stress; ROS, reactive oxygen species; NLRP3, NOD-like receptor thermal protein domain-associated protein 3; JNK1/2, c-Jun N-terminal kinase 1/2; TLR, Toll-like receptor; TGR5, Takeda G protein-coupled receptor 5; GPR41/43, G protein-coupled receptor 41/43.

In the primary stages of atherosclerosis, endothelial cells, located in the outermost layer between the blood and arterial intima, have a vital role in regulating vascular homeostasis and exert various protective effects on blood vessels. When there is a disruption in permeability, endothelial cells can become damaged, which enables increased entry of circulating low-density lipoprotein (LDL) into the body, particularly in the presence of a high-fat diet or high blood pressure. These LDLs stimulate monocytes to differentiate into macrophages and commence the expression of scavenger receptors like CD36 and SRA1, enabling them to uptake oxidized lipids. Initially, this process is advantageous; however, as lipid accumulation progresses, foamy macrophages become highly inflammatory and release chemotactic signals to attract additional monocytes, thereby exacerbating the inflammatory milieu rich in lipids (72). In the context of atherosclerosis, alterations in the gut microbiota can potentially induce systemic inflammation and trigger the polarization of macrophages into a proinflammatory M1 phenotype. As depicted in Fig. 3, beneficial bacteria residing in the gut have the ability to modulate the overall inflammatory status. According to Fatkhullina et al., inhibiting the IL-23-IL-22 signaling pathway results in the dysfunction of the intestinal barrier, proliferation of bacteria with atherogenic properties, and the production of metabolites that induce macrophage activation and contribute to the development of atherosclerosis. The equilibrium between IL-23 and IL-22 plays a role in shaping the gut microbiota by controlling the production of antimicrobial peptides and suppressing the growth of semi-invasive bacterial species that exhibit atherogenic properties (73). The RCT pathway aids in the elimination of cholesterol from cells present in atherosclerotic plaques, transports it into the bloodstream, and ultimately excretes it in feces. Activation of the liver X receptor (LXR) by macrophages or foam cells can enhance the efflux of free cholesterol, thereby preventing cholesterol toxicity. This activation leads to an increase in the expressions of cholesterol transporters like ABCA1 and ABCG1. Lipopolysaccharide (LPS), an endotoxin produced by gut microbes, can induce chronic low-grade inflammation. Even low doses of LPS have been observed to decrease the expression of proteins involved in cholesterol efflux, such as ABCA1/ABCG1 and SR-B1, which are involved in cholesterol efflux, in mouse macrophages (74). Bacterial LPS triggers the production of matrix metalloproteinase-9 (MMP-9) by activating toll-like receptor 2/4 (TLR2/4) in macrophages, VSMCs, and endothelial cells (Table 2). This activation contributes to vascular remodeling associated with atherosclerosis and the instability of plaques (75, 76). The activation of TLR2-/4-dependent stimulation can be effectively inhibited by liver X receptor alpha (LXRα), thereby blocking the upregulation of MMP-9 in LPS-stimulated macrophages (77).

**TABLE 2:** Evidence that microbial metabolites influence atherosclerosis^*a*^

Name	Experimental subject	Signal path	Influence index^*b*^	References
TMAO	HAECs	NF-κB	↑ COX-2, IL-6, E-selectin, and ICAM-1	
TMAO	Wild-type mice	NLRP3	↑ IL-1β; caspase-1 activity; CAEC	(78)
TMAO	HUVECs	NLRP3/SIRT3-SOD2-mtROS	Activation of caspase-1 p20 expression and caspase-1; ROS; inhibition of SOD2 and SIRT3 expression	(79)
TMAO	HCAECs	NF-κB	Promoted the expression of TF (but not TF pathway inhibitors) and thrombin production in HCAECs	(80)
TMAO	ECs and HUVECs	SDHB/ROS	Pyroptosis, ROS, and SDHB was increased; overexpression of SDHB in HUVECs enhanced cell apoptosis	(81)
TMAO	ECs		↓ NO, cell viability and tube formation; ↑ ROS	(82)
TMAO	HUVECs	ROS-TXNIP-NLRP3	Trigger oxidative stress and activate inflammasome, IL-β, and IL-18; inhibition of the expression of eNOS and NO	(83)
TMAO	HUVECs	PKC/NF-κB/VCAM-1	Suppressed HUVEC migration; up-regulated VCAM-1 expression; promoted monocyte adherence and activated PKC and p-NF-κB expression	(84)
TMAO	EPCs		↑ hsCRP and IL-1β, increased oxidative stress and inhibited EPC function	(85)
Butyric or propionic acid	HUVECs		↑ ICAM-1 and E-selectin	(86)
SCFAs	Asc^+/+^Mice/ECs	NLRP3	Both acetic acid and propionic acid can significantly promote the formation and activation of the NLRP3 inflammasome, while butyric acid has the opposite effect.	(87)
CDCA	HUVECs	VEGF/MMP9	↑ VEGFR1 and VEGFR2, MMP9 ↓ VE-Cadherin ↑ LCA	(88)
UDCA	ECs	RAGE and NF-κB	↓ ER stress ↓ RAGE expression ↓ NF-κB and ROS	(89)
UDCA	ECs	ER stress/ IRE1	↓ Reduced ER stress in ECs, the expressions of XBP-1 and CHOP were decreased in ECs and adhesion molecules	(90)
Taurine	HUVEC		Reduced ROS, ICAM-1, IC calcium, TNF-α and ADMA; increasing NO expression	(91)
Melatonin	HAECs	MEG3/miR-223/NLRP3	Blocking pyroptosis of HAECS-related genes (NLRP3, ASC, cleaved caspase1, NF-κB/GSDMD, GSDMD N-termini, IL-1β, and IL-18)	(92)
Melatonin	HUVECs	RORα	↑ IL-1β, IFN-γ, TNF-α, ROS, ICAM-1 and VCAM-1, NO, and the expression of SOD1, GPX1, and CAT	(93)
LPS and indoxyl sulfate	HUVECs		↑ ROS, E-selectin, and MCP-1	(94)
*Lactobacillus gasseri* SBT2055, *Lactobacillus paracasei*, *Lactobacillus plantarum* CLP-0611, and *Lactobacillus brevis* G-101	Macrophage	NA	↓ Number of macrophages and M1/M2 ratio in mice	(95–98)
Butyrate	Macrophage	FFA3 receptor	↓ iNOS, TNFα, MCP-1, and IL-6	(99)
SCFAs	Macrophage	Activate GPR41 and GPR43 expression	↓ IL-8	(100)
1.0 mmol/ L butyrate	Human macrophage cell		↑ ROS, caspase-1, and IL-1 β	(101)
Butyrate	Mice peritoneal macrophages		↓ ROS and NO	(102)
TMAO	Macrophage		Downregulated expression of ABCA1	(103)
TMAO	Apoe^–/^– mice, macrophage RAW264.7	MAPK/JNK	Macrophage recruitment, CD36 and proinflammatory cytokine expression *In vitro*, ↑ macrophage migration, TNF-α, IL-6, and ICAM1 expression ↑ Ox-LDL-induced CD36 expression and foam cell formation	(80)
*Lactobacillus acidophilus* K301	Macrophage	LXR	Upregulated expression of ABCA1 and ABCG1, promoting RCT	(104)
CDCA	Human monocytes and macrophages	TGR5/NF-kB	↓ Phagocytic activity and proinflammatory cytokines	(105)
BAs	Macrophages	TGR5/NLRP- 3	↓ IL-1 and IL-18	(106)
UDCA	Macrophages		↓ Expression of RAGE and proinflammatory cytokines; upregulated expression of ABCA1 and ABCG1	(89)

^*a*^ SCFAs, short chain fatty acids; BAs, bile acids; TMAO, trimethylamino oxide; LPS, lipopolysaccharide; CDCA, chenodeoxycholic acid; UDCA, ursodesoxycholic acid; IL-1β, interleukin-1β; IL-6, interleukin-6; IL-18, interleukin-18; IL-1, interleukin-1; TNF-α, tumor necrosis factor-α; hs-CRP, high-sensitivity C-reactive protein; VCAM-1, vascular cell adhesion molecule-1; MMP9, matrix metalloprotein9; SOD1, superoxide dismutase1; GPX1, glutathione peroxidase 1; CAT, catalase; CD36, cluster of differentiation 36; NF-κB, nuclear factor-k-gene binding; ER stress, endoplasmic reticulum stress; ROS, reactive oxygen species; NLRP3, NOD-like receptor thermal protein domain-associated protein 3; JNK, c-Jun N-terminal kinase; TLR, toll-like receptor; TGR5, Takeda G protein-coupled receptor 5; GPR41/43, G protein-coupled receptor 41/43.RCT, reverse cholesterol transport; MCP-1, monocytechemoattractant protein-1; ABCA1, ATP-binding cassette transporter A1; ABCG1, ATP-binding cassette sub-family G member 1; RAGE, receptor for advanced glycation end-product; ICAM1, intercellular cell adhesion molecule-1; MAPK, mitogen-activated protein kinase; LXR, liver X receptor; iNOS, nitric oxide synthase; NO, nitric oxide; FFA3, free fatty acid receptor 3; HAECs, human aortic endothelial cells; RORα nuclear receptor retinoid-related orphan receptor alpha; ASC, apoptosis-associated speck-like protein containing a caspase-recruitment domain; GSDMD, gasdermin domain-containing protein; MEG3, maternally expressed gene 3; ADMA, asymmetric dimethylarginine; XBP-1, X-box binding protein-1; CHOP, CEBP - homologous protein; ECs, endothelial cells; IRE1, inositol-requiring enzyme 1; LCA, lithocholic acid; PKC, protein kinase C; VEGFR1, vascular endothelial growth factor 1; VEGFR2, vascular endothelial growth factor 2, EPCs, endothelial progenitor cells; eNOS, endothelial nitric oxide synthases; TXNIP, thioredoxin-interacting protein; SDHB, succinate dehydrogenase complex subunit B; HUVECs, human umbilical vein endothelial cells; HCAECs, human primary coronary endothelial cells; CAEC, cell permeability of carotid endothelial cells; HAECs, human arterial endothelial cells; COX-2, cyclooxygenase-2.

^*b*^ The downward arrow indicates a decrease, while the upward arrow indicates an increase.

Within the gastrointestinal tract, microorganisms have the capability to convert trimethylamine (TMA) found abundantly in choline, phosphatidylcholine, and carnitine into trimethylamine N-oxide (TMAO). TMAO serves as a crucial molecule that activates inflammatory pathways impacting the blood vessel wall. This, in turn, promotes the development of atherosclerosis (107). TMAO has the ability to induce oxidative stress and activate the NLRP3 inflammasome, leading to the release of pro-inflammatory cytokines. Furthermore, TMAO can reduce the levels of endothelial nitric oxide synthase (eNOS) and impair nitric oxide (NO) production (83). Endothelial dysfunction occurs as a consequence of intracellular inflammation and the oxidative stress response, leading to the production of ROS and a decrease in NO levels. This process ultimately impairs the function of circulating EPCs. Consequently, there is increasing evidence pointing to the impact of TMAO on endothelial cells involved in the development of atherosclerosis (85) (Table 2). These findings indicate that TMAO plays a significant role as a novel facilitator of endothelial dysfunction. More than 30 years ago, researchers discovered that extracts from *Proteus mirabilis* bacteria could break the C-N bond of choline, resulting in the formation of TMA and acetaldehyde (108), In addition, it has been observed that *Escherichia fergusonii* also exhibits TMA lyase activity. Wang et al. (109) discovered a strong positive correlation between *Clostridia* and *Ruminococcus* bacteria with plasma TMA and TMAO levels, as well as the area of atherosclerotic lesions. Furthermore, they found a significant negative correlation between the proportion of *S24-7* bacteria (belonging to the Bacteroidetes family) and the size of atherosclerotic plaques, as well as the level of TMA. Previous studies have reported the ability of *Clostridium* bacteria to cleave choline (110). As a result, the strategy of targeting different microbial TMA lyases to inhibit the production of trimethylamine N-oxide (TMAO) and the formation of foam cells has emerged as a promising approach to slow down the progression of atherosclerotic lesions. This approach holds potential as a novel therapeutic target.

SCFAs are advantageous products derived from intestinal microbes and have demonstrated anti-atherogenic effects. They possess the ability to decrease systemic inflammation, enhance endothelial cell functionality, and fortify the intestinal barrier (111, 112). SCFA-producing bacteria mainly include *Akkermansia muciniphilia*, Bacteroides spp., *Bifidobacterium* spp., *Prevotella* spp., *Ruminococcus* spp*., Phascolarctobacterium succinatutens, Roseburia inulinivorans*, *Ruminocossus obeum*, *Roseburia inulinivorans*, *Ruminocossus obeum*, *Anaerostripes* spp., *Eubacterium hallii*, and *Faecalibacterium prausnitzii* (101). Studies have indicated that SCFAs are involved in preserving endothelial cell function and contribute to vasodilation. They can induce the dilation of resistant blood vessels through an endothelium-dependent mechanism by activating GPCR41 receptors (113). In *ApoE ^–/^*^–^ mice, oral administration of butyrate can decrease the expression of NADPH oxidase and iNOS, leading to a reduction in oxidative stress, atherosclerotic lesions, and the production of aortic superoxide ions. Furthermore, treatment with butyrate in Ea.hy926 cells has been shown to lower the levels of ox-LDL uptake, CD36, VCAM-1, TNF-α, and IL-1β/6 production, while increasing the production of IL-10 (102). In addition, butyrate can effectively inhibit diabetes-induced endothelial dysfunction and high-fat diet (HFD)-induced oxidative stress by activating the nuclear factor E2-related factor 2 (Nrf2) pathway (nuclear factor erythroid 2-related factor 2) (114, 115). SCFAs have been shown to regulate immune homeostasis by functioning as inhibitors of histone deacetylase (HDAC). Butyrate, in particular, has been demonstrated to promote cholesterol efflux. Both *in vivo* and in *vitro* studies have revealed that butyrate significantly induces the expression of ATP-binding cassette transporter A1 (ABCA1), thereby enhancing cholesterol efflux. These findings suggest that butyrate holds promise as a potential therapeutic strategy for preventing atherosclerosis (116).

Consequently, a promising approach to slow down the advancement of atherosclerotic lesions involves targeting various microbial TMA lyases to inhibit the synthesis of TMAO and the formation of foam cells (102). However, further clinical and mechanistic investigations are required to gain deeper insights into the protective effects of butyrate on endothelial cells. Additionally, BAs are essential bioactive compounds that play a role in regulating homeostasis of triglycerides, cholesterol, and glucose. Bile acids can function as activators or inhibitors of receptor-mediated signal transduction. There they are deconjugated by microbial bile salt hydrolase, mainly from *Bifidobacteria*, *Clostridia,* and *Bacteroidetes*, to secondary bile acids, which are reabsorbed into the circulation (117). It has been proposed that both SCFAs and BAs may modulate endothelial cells and exert an influence on atherosclerosis (Fig. 3

#### Effects of gut microbiota in the mid-term stages of atherosclerosis

Vascular smooth muscle cells are the primary cell type found in the mid-term stages of atherosclerotic plaques. These VSMCs play a critical role in AS as they serve as a significant source of macrophages. Macrophages derived from VSMCs actively contribute to the progression from the pre-atherosclerotic (preAS) phase to the formation of fibrotic plaques. Endothelial injury triggers the proliferation and migration of VSMCs toward the arterial intima. Additionally, VSMCs secrete a substantial number of adventitial cells, which contributes to the diffuse thickening of the inner layer of the blood vessel wall (118). During the early stages of AS, VSMCs undergo proliferation and uptake of oxidized low-density lipoprotein (oxLDL), which triggers the activation of several proinflammatory genes. This upregulates markers associated with activated macrophages. To switch to a macrophage-like phenotype, integrin beta 3 (Itgb3) is downregulated, leading to the upregulation of TLR4, CD36, CD68, and MAC-2 (119). Consequently, foam cells are formed, contributing to the pathological thickening of the inner vessel wall, known as pathological intimal thickening (PIT). DCA (deoxycholic acid) was found to mediate the proliferation and migration of VSMCs through the JNK pathway (120). On the other hand, UDCA activates the Mir-21/PTEN/AKT/mTOR pathway to inhibit excessive migration and proliferation of VSMCs, consequently limiting intimal hyperplasia (121). Additionally, UDCA also inhibits the expression of iNOS (inducible nitric oxide synthase) and the production of nitric oxide (NO) (122). Taurine has been shown to possess antiproliferative and antioxidant properties, thereby exerting a protective effect on VSMCs (123). SCFAs impact the progression of atherosclerosis by activating the GPR41 receptor in VSMCs, which subsequently influences blood pressure regulation (124). Furthermore, there is evidence suggesting that TMAO promotes calcification in VSMCs of both rats and humans in a dose-dependent manner. This effect is mediated by the activation of the NLRP3 inflammasome and NF-κB signaling pathway (125).

#### Effects of gut microbiota in the late stages of atherosclerosis

In the advanced stage, when VSMCs undergo apoptosis or cell death, IL-1β is released, and lipid accumulation occurs, resulting in the formation of extensive fibrous plaques. At this point, activated T cells act as antigens and secrete inflammatory mediators, exacerbating plaque instability and further progression of the disease (126). T lymphocytes can be classified into different subgroups based on their immunophenotype. Among these subsets are helper T (Th) cells and regulatory T2 (Treg) cells. Th1 cells play a role in promoting inflammatory responses by releasing proinflammatory cytokines, including IFN-γ, TNF-α, and TNF-β (127). Treg cells possess the ability to suppress macrophage phagocytosis of ox-LDL through an interaction with a scavenger receptor. Additionally, Treg cells possess the ability to suppress the immune response of DCs, Th1, Th2, and Th17 cells, while simultaneously enhancing the expressions of TGF-β, IL-10, and IL-5. These cells play a crucial role in maintaining the host’s immune and inflammatory response homeostasis. Consequently, T cells can serve as either positive or negative regulators, actively participating in the formation and perpetuation of atherosclerotic plaques (128). Different subsets of CD4 +T cells can be found accumulating within human atherosclerotic plaques (129). Depending on the specific subsets, CD4 +T cells can exhibit either protective or pathogenic characteristics by acquiring a Th1 cell phenotype. In a study conducted by Kim et al., it was observed that the administration of *Lactobacillus rhamnosus* Lcr35 led to an increase in the population of CD4 +CD25+Foxp3+ Treg cells population in the spleen and mesenteric lymph nodes of mice (130–132). Therefore, enhancing the response of Treg cells has been shown to improve atherosclerotic lesions, as demonstrated in a study by Yamashita et al. (133). In addition, *Lactobacillus casei* Shirota reduced the Th2 cell phenotype in the same mouse model, while *Lactobacillus plantarum* WCFS1 enhanced the Th2 cell phenotype (134). It has been observed that butyrate and low concentrations of propionate strongly promote Treg cell differentiation in the mouse colon. However, at a concentration of 1 mmol/L, butyrate can induce Th17 cells and exacerbate inflammation by stimulating the production of IL-23 in DCs (135, 136). The activity of indoleamine 2,3-dioxygenase is renowned for its ability to dampen the immune response of effector T cells and stimulate the activation of regulatory T cells. However, deletion or inhibition of indoleamine 2,3-dioxygenase has been shown to improve insulin sensitivity, preserve the gut mucosal barrier, and reduce endotoxemia and chronic inflammation, as evidenced by studies conducted by Laurans et al. (137). These findings indicate that different strains of bacteria and their metabolites play distinct roles in immune responses when intervened upon. Further research is needed to better understand the interaction between T cells and the microbiota.

B cells are found within the arterial adventitia, and their primary function involves the recognition and processing of antigens, which eventually leads to their differentiation into plasma cells in the advanced stages of atherosclerosis. These plasma cells are responsible for producing antibodies and cytokines (138). However, when compared to the inflammation-promoting abilities of macrophages and specific T-cell subsets during atherosclerosis, B cells have a relatively smaller impact on the inflammatory processes occurring in the blood vessel wall (139, 140). The involvement of B cells in the progression of atherosclerosis is complex and still not fully comprehended. They may have a complex impact through their production of antibodies, but the precise mechanisms involved require further investigation (141). Recent studies have consistently demonstrated the protective role of B1 cell-derived natural IgM antibodies against atherosclerosis (142, 143). These IgM antibodies have the ability to bind to oxidative motifs present in LDL, specifically targeting phosphocholine head groups on cell wall polysaccharides of pathogens like *Streptococcus pneumoniae* and apoptotic cells. *In vitro* experiments have further revealed that native IgM effectively blocks the uptake of oxidized LDL by macrophages (144). On the other hand, the B2 cell response may contribute to the development of atherosclerosis by providing support to proatherogenic T cells (145). Similar to other immune cells, B cells undergo changes in the expression patterns of TLRs. Intestinal commensal microorganisms have been identified as significant sources of antigens that activate specific splenic B2 cells associated with atherosclerosis. Commensal microbes or commensal microbe-derived LPS or peptidoglycan can be selectively recognized by the host’s innate immune TLRs (146). In cases of hyperlipidemia, gut commensal microflora triggers the activation of B2 cells in the spleen through the TLR signaling pathway. Consequently, these activated B2 cells modify antigen presentation, leading to increased levels of circulating IgG and IgG3. Additionally, they produce cytokines and chemokines, which may contribute to the advancement of atherosclerosis (147). These findings demonstrate a causal relationship between B2 cells and microbially induced atherosclerosis and may explain why lipid control alone is not an effective preventive treatment in some patients with atherosclerosis. Apart from B1 and B2 cells, regulatory B cells (Bregs) also play a role in the regulation of atherosclerosis. Bregs produce immunosuppressive cytokines such as IL-10 and TGF-β (148). The gut microbiota has a direct impact on the development, activation, and regulation of health and disease in Bregs. Studies have shown that intestinal microbiota-derived butyrate induces the differentiation of Bregs cells to produce IL-10, and whether microbiota derived metabolites can regulate the inhibitory function of Breg remains unclear (149). Therefore, targeting activated B2 cells or promoting the production of atherosclerotic protective B1 cells and Bregs through gut microbes and metabolites may be a potential treatment for atherosclerosis

### Remodeling the gut microbiome improves atherosclerosis

**Fig 4:**
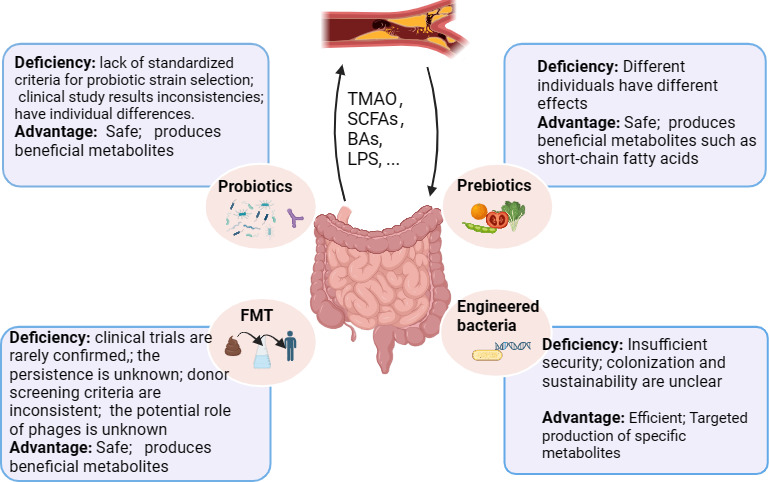
Strategies for reshaping intestinal microbiota to intervene in atherosclerosis. SCFAs, short-chain fatty acids; TMAO, trimethylamino oxide; LPS, lipopolysaccharide; BAs, bile acids.

As stated previously, patients with atherosclerosis exhibit dysbiosis in their intestinal microbiome, and reshaping the gut microbiome has emerged as a novel strategy to improve the disease process. Dietary interventions are commonly employed to regulate the gut microbiome. In recent years, the utilization of probiotics, prebiotics, FMT, and engineered bacteria has become a new approach for reshaping the gut microbiome (Fig. 4).

#### Probiotics

Probiotics refer to a group of live microorganisms that are consumed orally and can exert beneficial effects within the human body. Probiotics have the functions of diarrhea prevention (150, 151), blood pressure reduction (152), antioxidant (153), constipation prevention (154) and inhibiting tumor growth (155), and so on. Liang et al. discovered that *Lactobacillus plantarum* strain ZDY04 significantly reduces the levels of TMAO and TMA in the serum of ApoE^−/−^ mice. Additionally, it reshapes the gut microbiota (156). Probiotics exert antimicrobial effects and reduce the production of harmful metabolites in the body. A study has shown that the lipoteichoic acid produced by inhibits the production of the pro-inflammatory factor LPS and the expressions of ICAM-I, VCAM-1, and E-selectin in HUVEC cells. Furthermore, it inhibits the production of MMP-9 and CD31 in ApoE–/– mice (157).

The benefits of probiotics are specific to certain diseases as well as specific to particular strains of bacteria (158). However, there are still several challenges in the field. First, there is a lack of standardized criteria for probiotic strain selection, leading to variations in the evaluation of their therapeutic efficacy. Second, inconsistencies between basic research and clinical study results hinder the translation of promising findings into clinical applications. Third, the mechanisms by which probiotics improve atherosclerosis remain unclear. Additionally, the gut microbiota exhibits spatial variation along the different regions of the intestine, but sampling difficulties often result in the use of fecal samples as representatives of the entire gut lumen. Localized sampling would contribute to a more accurate understanding of the gut’s biology. Therefore, well-designed experiments are needed to investigate the composition of the gut microbiota before and after interventions. Ideally, metagenomic sequencing (rather than 16S rRNA sequencing) should be employed to provide species-level resolution for compositional data.

#### Prebiotics

Prebiotics and dietary interventions indirectly target the gut microbiota composition. Prebiotics selectively stimulate specific microbes in the colon. Prebiotics are typically fibers, although not all fibers are prebiotics (159). Prebiotics have been shown to stimulate the growth of SCFA-producing microbes such as *Bifidobacterium* and *Lactobacillus*. SCFAs regulate oxidative stress and improve inflammatory levels, impacting disease progression. A study involving 12 patients demonstrated that inulin (a long-chain prebiotic) significantly decreased serum triglycerides and low-density lipoprotein cholesterol, while increasing serum high-density lipoprotein cholesterol levels (160). Furthermore, a mixture of inulin/fructooligosaccharides (10 grams per day) stimulates the growth of *Bifidobacterium (*161). β-glucan is another prebiotic that has a significant impact on reducing cholesterol levels and maintaining blood glucose stability. After 2 months of consuming beta-glucan, 26 healthy volunteers exhibited significant reductions in LDL-cholesterol and total cholesterol levels. Additionally, endothelial function in healthy individuals improved, mainly attributed to the production of beneficial short-chain fatty acids by the gut microbiota, providing cardioprotective effects (162). Patients with atherosclerosis often exhibit dysbiosis in their gut microbiota. Further metagenomic analysis of the fecal microbiome’s functional capacity reveals an enrichment of genes encoding for peptidoglycan synthesis and a deficiency of plant sterol dehydrogenases in symptomatic atherosclerosis patients. This suggests that dysbiosis in the gut microbiota can lead to altered intestinal function (42). Therefore, prebiotics contribute to alleviating disease progression by increasing the abundance of beneficial bacteria, producing beneficial metabolites, and reducing the abundance of harmful bacteria.

#### FMT

FMT has been demonstrated to be effective with limited side effects (163). Hao et al. utilized FMT from donors to host mice, demonstrating a reduction in atherosclerosis, systemic inflammatory response, as well as alterations in gut microbiota composition and associated metabolites (164). In a study, FMT significantly impacted the gut microbiota of both CTRP9-KO and WT mice, with all transplant recipients acquiring the donor mouse’s gut microbiota. Following transplantation, CTRP9-KO mice exhibited reduced atherosclerotic lesions, indicating that FMT can reshape the gut microbiota and alleviate atherosclerosis (165). Moreover, bacteriophages—which have long been understudied but are now known to play important roles in the microbiome—have been demonstrated to transfer from the donor to host via FMT with uncertain implications (166). In population cohort studies, the FMT trial has targeted cardiovascular risk by transplanting lean, plant-based diet donor microbiota into meat-consuming individuals with metabolic syndrome to reduce TMAO levels (167). Although there were changes observed in the composition of the gut microbiota, TMAO levels did not show any significant changes following the intervention. Other FMT trials targeting obesity and metabolic syndrome have also indicated minimal and transient effects of FMT on microbial composition and glucose metabolism, emphasizing the importance of pre-screening to select recipients who are most likely to respond (168). Although data on the role of FMT in individuals with features of metabolic syndrome are limited, a study randomized 18 treatment-naïve male patients with metabolic syndrome to receive a single duodenal infusion from lean healthy donor-derived allogeneic FMT or autologous FMT (their own feces as placebo). Six weeks post-infusion, peripheral blood insulin sensitivity improved only in recipients of microbiota from healthy donor subjects. This change was also accompanied by increased bacterial diversity (169). Interestingly, a study involving 237 patients (42 with multiple sclerosis and 195 without multiple sclerosis) found that Washed Microbiota Transplantation (WMT) significantly restored gut microbiota homeostasis in multiple sclerosis patients, improving blood sugar, lipid levels, blood pressure, and BMI (170), suggesting the therapeutic potential of FMT in the treatment of metabolic diseases. However, optimal FMT methods, including donor selection, screening and preparation, participant numbers, gender considerations, administration via upper gastrointestinal, enema, or colonoscopy routes, as well as short-term and long-term monitoring of adverse reactions and therapeutic outcomes, present new challenges that require further determination (171–173).

#### Engineered bacteria

Engineered bacteria are microorganisms modified through genetic engineering to achieve specific functions and traits, leading to desired gene expression and metabolic outcomes (174). Currently, therapeutic bacteria can produce various engineered products, including small molecules, vaccine antigens, enzymes, cytokines, and antibodies. These bacteria are tailored to generate beneficial metabolites with enhanced yields and added safety features, such as passive containment systems. Additionally, the controlled release of these metabolites to the distal small intestine or colon may minimize off-target effects (175). In the development of atherosclerosis, researchers used strains of *Lactococcus lactis* NZ9000 and *Escherichia coli* Nissle 1917 to produce the heat shock protein −65 n-acylphosphatidylethanolamine (npe-pld). These strains were administered orally to mice to help mitigate the atherogenic process (176, 177). Research has indicated that mice lacking APE-PLD in the intestinal epithelium (Napepld mice) exhibit increased appetite, obesity, and steatosis following an initial high-fat diet (HFD). Additionally, these mice show abnormalities in hypothalamic Pomc neurons and altered levels of NAE and 2-acylglycerol in both the gut and plasma (178). Undoubtedly, engineered bacteria have emerged as a promising new method for treating many diseases. There is still a need to identify more potential targets for therapeutic or beneficial metabolites or small molecules related to atherosclerosis. Through genetic modifications, these targets can be stably colonized and expressed in the gut, offering a new strategy to mitigate disease progression. During clinical translation, it is essential to assess biological protection and safety. BCG, an attenuated *Mycobacterium bovis* strain used for bladder cancer treatment, serves as a benchmark for FDA-approved bacterial cancer therapies. Besides BCG, other individual attenuated or engineered strains with therapeutic payloads are also being evaluated in patients (179). Therefore, the application of engineered bacteria in clinical trials should be fully attenuated to ensure safety.

Gut bacteria and their metabolites can alter the physicochemical properties of nanoparticles, while nanoparticles can influence the metabolism of bacteria and their interaction with intestinal lymphoid tissue (180). Based on the interactions between nanoparticles and gut microbiota and the use of nanotechnology in probiotic delivery and release, there is potential to apply these approaches to target gut microbiota in the treatment of atherosclerosis. However, the clinical application of nanoparticle-based products still requires further biological research (181). As a burgeoning interdisciplinary field, nanomaterials present exciting possibilities for treating atherosclerosis. However, further clinical and cohort studies are needed to fully understand the relationship between gut microbiota, nanomedicine, and atherosclerosis. Additionally, exploring gene editing techniques to introduce fluorescent proteins into bacteria or identifying bacteria-specific molecular targets for visual tracking could help elucidate bacterial pathways and targets.

### Conclusion

Although our understanding of how the gut microbiota influences cardiovascular diseases is still in its early stages, the rapid emergence of new discoveries is impressive. Novel therapeutic approaches targeting the gut microbiota for the treatment and prevention of cardiovascular diseases represent an exciting research field. Developing non-lethal microbial inhibitors targeted at specific pathways, with limited systemic exposure in the host, is just one of the new and promising therapeutic approaches. However, there is a substantial body of evidence indicating that metabolites generated by microorganisms exert an influence on disease processes. Notable metabolites in this regard include butyric acid, bile acids (BAs), trimethylamine-N-oxide (TMAO), lipopolysaccharides (LPS), as well as specific thiols and indole derivatives. These microorganisms and their metabolites interact with cells involved in atherosclerosis, making them promising therapeutic targets for intervention and influencing disease progression. Prebiotics and biologically active substances such as polyphenolic compounds (e.g. catechin, quercetin-3-glucoside, α-lipoic acid, ellagic acid, resveratrol, inulin, sorbitol, and uric acid) have been shown to improve gut dysbiosis and prevent atherosclerosis. FMT has also been validated as an adjunctive therapy. However, it is important to identify which microbial genes are associated with atherosclerosis and the pathways affected by their metabolites. Finally, through cloning and expression of these enzymes and genes, they may one day be used to “reprogram” the microbiota, altering their functional outputs to benefit the host. Like any therapeutic approach, large-scale prospective intervention studies are needed to validate new gut microbiome-targeted therapies.
